# Creative Flow as a Unique Cognitive Process

**DOI:** 10.3389/fpsyg.2017.01348

**Published:** 2017-08-08

**Authors:** Charlotte L. Doyle

**Affiliations:** Psychology Department, Sarah Lawrence College Bronxville, NY, United States

**Keywords:** cognition, flow, dual process theories, divergent thinking, convergent thinking, insight, incubation, intuition

## Introduction

The concept of flow, an experience of total engagement in an activity, was introduced into psychology by Csikszentmihalyi (1975) based primarily on first-hand accounts in a variety of domains. He found examples in physical activities such as rock climbing, sports (where it is also known as *being in the zone*), games such as chess, religious rituals, occupational activities such as surgery, and creating in the arts (creative flow). Csikszentmihalyi (1999) described the elements of the flow experience this way: The sense of having stepped out of the routines of everyday life into a different reality (See also Schutz, 1945), clear goals every step of the way, immediate feedback, effortless attention, action and awareness merged, balance between skill and challenge, time distortion, and spontaneity. These properties are cognitive; they are relevant to the study of problem representation (Newell et al., 1958; Pretz et al., 2003), automatic vs. controlled cognitive processes (Schneider and Shiffrin, 1977; Meier et al., 2003), time perception (Zakay and Block, 1996), and modes of cognition (Evans, 2008).

Csikszentmihalyi (1999) also noted common cognitive contents no longer present; no distractions such as what Smallwood and Schooler (2006) called mind-wandering, no fears of failure (Clark et al., 1956), none of the usual self-consciousness of everyday life (Schutz, 1945). Csikszentmihalyi also recognized a paradox with respect to control: Flow feels effortless with no conscious sense of controlling what emerges, but, given flow's characteristics, he assumed that “one has to be in control of the activity in order to experience it” (Csikszentmihalyi, 1999, p. 825).

Flow has been a robust subject for research and theorizing (Engeser, 2012; Harmat et al., 2016). The general assumption has been that the features of flow did not differ from one domain to another (see Cseh, 2016, for an exception). Experimental research has been centered on activities that can be easily observed, controlled and varied in the laboratory such as computer gaming. (See, for example, Klasen et al., 2012). That study confirmed the elements of flow as Csikszentmihalyi (1975) first described them.

Among the domains for which a flow experience has been described are those in the creative arts—writing (Perry, 2009), painting (Banfield and Burgess, 2013), and musical composition (Csikszentmihalyi, 1975). Evidence is emerging that the flow experienced by those creating in domains of the arts (creative flow), while sharing most of the properties Csikszentmihalyi wrote about, also has a few properties that distinguish it from flow in other domains. Interviews with visual artists suggested that in this domain, goals, which are part of problem representations, are not clear (Mace, 1997). One artist told her, “You really don't know where you are going” (p. 274). In another interview study, Cseh (2017) concluded that clear goals, sense of control, and unambiguous feedback were not typically part of fine artists' flow experiences.

Doyle (1998) noted another feature of creative flow: that what emerges is often surprising to the maker. One writer told about writing a story about a man who, in the scene being written, was lying in bed with his wife. As he was speaking of his son, his wife interrupted and said, “Is it happening again…Jimmy's not real” (Doyle, 1998, p. 33). The author, who had assumed the son was real, was startled out of flow with the unexpected realization that the son the husband had been speaking of was only his delusion.

Furthermore, creative flow involves meaning-making, as Csikszentmihalyi's own interview with a writer suggested.

It's just an extended present…in which you are making meaning. And dismantling meaning and remaking it (Csikszentmihalyi, 1996, p. 121).

The meaning making happens in a rush as the term *flow* implies. A composer gave this description when asked how it felt when his work was going well:

“…My hand seems devoid of myself, and I have nothing to do with what is happening. I just sit there watching in a state of awe and wonderment. And the music just flows out by itself.” (Csikszentmihalyi, 1975, p. 44).

Thus, unclear goals, uncertain feedback, the possibility of surprise, and rapid meaning-making are cognitive properties of creative flow along with properties shared with other flow domains: taking place in a reality outside the everyday, effortless attention, action and awareness merged, balance between skill and challenge, time distortion, spontaneity, non-distractibility and no self-consciousness or personal fears.

Descriptions of creative flow are based on interviews; probably for this reason, its features have not been considered in laboratory-based accounts of cognition. Yet, as Ward (2001) proposed, important advances can come from a convergence approach, drawing on both laboratory research and first-hand accounts. This article looks at the features of creative flow in relation to other cognitive phenomena. The article argues that this analysis will broaden and complicate understanding of the possibilities of cognition. The complexities emerge as the properties of creative flow are considered in relation to those of type 1 vs. type 2 cognition; convergent vs. divergent thinking, incubation and insight, all topics that have been the subject of extensive laboratory research and theorizing.

## Dual process theories

Dual process theories of cognition arose in conjunction with studies of reasoning, decision-making, and social cognition. Though different theorists (Smith and DeCoster, 2000; Epstein, 2003; Evans, 2008; Stanovich, 2009; Kahneman, 2011; Strack and Deutsch, 2012) put forward versions which vary in a few features, there are common threads. All distinguished between intuitive thinking (Type 1), characterized as fast, automatic, and high capacity, vs. deliberative or reflective thinking which is slow, controlled, and low capacity (Type 2). Evans (2014) conceptualized the Type 2 features as enabling two other properties, hypothetical thinking and cognitive decoupling–keeping representations decoupled from the actual world.

Theorists have explored the relation between the thinking types and creative process phases other than flow. Allen and Thomas (2011) investigated their role in problem-finding, conceptualization, incubation, illumination, verification, and dissemination. They concluded that Type 1 thinking is typical of incubation, but that the other phases involve both in some proportion. Similarly, Sowden et al. (2015), looking at the idea generating and evaluation phases of the creative process, suggested that shifts between Type 1 and Type 2 thinking are likely in both phases.

Creative flow has properties different from the other phases. Rather than exhibiting shifts, it, as a single phenomenon, includes some characteristics from each of the two types. It is intuitive and comes quickly (Type 1). It is not experienced as controlled (Type 1) though Csikszentmihalyi proposed it has to be (Type 2). Creative flow is obviously decoupled from the actual world (Type 2), as it takes place in a sphere other than everyday reality (Sessions, 1952; Doyle, 1998; Csikszentmihalyi, 1999).

## Convergent and divergent thinking

Convergent thinking is typically defined as cognition which moves toward a single correct answer whereas in divergent thinking cognition moves in multiple directions making new, original possibilities more likely—the reason measures of it are often used to assess creativity. Research has shown that the more original associations happen late in the associative stream after the first more conventional associations come to mind (Mednick, 1962; Milgram and Rabkin, 1980). Divergent thinking has been linked to defocusing (Gabora, 2010), with associative rather than rule-governed processes (Gabora, 2010; Goldschmidt, 2016), and cortical hypofrontality (Yoruk and Runco, 2014).

Like divergent thinking, creative flow results in something new and original and has been associated with hypofrontality (Dietrich, 2004; Limb and Braun, 2008), yet unlike divergent thinking, the originality comes quickly, and a meaningful, rule-governed structure emerges. Like convergent thinking, creative flow is focused, with distractions inhibited, involves rule-governed processes, and moves toward fulfillment of creative intentions.

## Insight and incubation

In the realm of problem solving, creative flow shares properties with both the insight and incubation phases. Insight has often been described as following a period of preparation and facilitated by a period of incubation (Sio and Ormerod, 2009) including mind-wandering from an intentioned task (Baird et al., 2012). Topolinski and Reber (2010) described insight's features: ideas come suddenly—“pop into the mind, abruptly and unexpectedly” (p. 402), and bring ease of processing after the solution is found. Creative flow shares the feature of coming to mind rather than found through effort, and may be surprising, but rather than a single idea which solves or restructures a prior problem, flow unfolds over time. The ease of processing is part of the emerging creation, not a process subsequent to it. The content of insight is typically an idea; creative flow typically comes embodied—the composer is at the keyboard; the painter, at the easel; the writer, at the computer or with pen in hand (See, for example, Banfield and Burgess, 2013). As Csikszentmihalyi (1999) wrote, idea, and action are fused.

Like incubation, flow often follows intentional preparation and is effortless—allowing the mind to go where it will without the control of executive functions. In incubation, the mind wanders away from the prior intentioned problem and is often referred to as task unrelated thought (Smallwood and Schooler, 2006); creative flow is focused on the prior problem—the unfulfilled creative intention. Yet flow results in something new, possibly unexpected, pointing to global access, a feature that has been suggested as one possible explanation for incubation (Sio and Ormerod, 2009).

Flow may happen anywhere in the sequence of phases of the creative process (Doyle, 2016). It may follow seamlessly after a period of effort or follow incubation. In other cases flow may be triggered by an insight—such as the idea of a new character coming to the writer, but the insight does not solve a problem; rather it gives a new direction to the subsequent flow rather than determining its course. In other cases, the intuitive flow itself leads to insight, the artist realizing the structure underlying the flow only on reflection afterward. For example, one writer told of writing a paragraph carried along by its rhythm and only in reflection realizing that it contained what was to be a major theme of the novel (Doyle, 1998). A similar phenomenon has been described in the classic problem solving literature; Anzai and Simon (1979) reported that, given the Tower of Hanoi problem, some subjects realized the pattern needed for solution only after carrying it out.

## Individual flow and group improvisation

The flow experienced by an individual artist shares properties with another creative activity—group improvisation as in theater or in jazz (Sawyer, 2003). Its properties include contingency—each participant's contribution triggering the next participant's response—and modifiability—subsequent events may change the meaning of what came before (Sawyer and DeZutter, 2009). Here, rather than the contingent actions involving several people, a single artist's brush strokes (Shahn, 1957), sentences (Doyle, 1998), or musical phrases (Csikszentmihalyi, 1975) are spontaneous responses to what came before. Ideally, what emerges in both creative flow and group improvisation has an underlying meaningful structure.

## Implications

Thinking in creative flow moves toward organization and meaning intuitively. This provides another clear example of a growing body of research (Dorfman et al., 1996; Betsch and Glöckner, 2010; Newman et al., 2017) demonstrating fast, intuitive thinking may be complex and organized. In creative flow, the pattern weaving, meaning-making, global reaching, integration seeking tendencies of the mind take place without conscious control, yet are in the service of an initial and yet unrealized creative intention. Creative flow shares some properties with both Type 1 and Type 2 cognition, both convergent and divergent thinking, with insight and incubation, but flow also has properties that distinguish it from each of these. Though flow is often only one phase of an extended creative process in which other phases have other properties, its unique features should be taken into account for a fuller understanding of the range of possibilities in the domain of cognition.

## Author contributions

The author confirms being the sole contributor of this work and approved it for publication.

### Conflict of interest statement

The author declares that the research was conducted in the absence of any commercial or financial relationships that could be construed as a potential conflict of interest.
